# Phlebectasia of Internal Jugular Vein- A Rare Differential Case of Neck Swelling With Review of Literature

**Published:** 2019-07

**Authors:** Amber Kesarwani, Amit Goyal, Amit Kumar

**Affiliations:** 1 *Department of Otorhinolaryngology, All India Institute of Medical Sciences, Jodhpur* *.*

**Keywords:** Jugular vein, Management, Phlebectasia

## Abstract

**Introduction:**

Internal jugular vein ectasia is a condition in which there is a dilatation of the internal jugular vein. A patient usually presents in the first decade with swelling in the neck, which aggravates in size while straining or coughing. This is a very rare condition and the chances of misdiagnosis are quite high. It is diagnosed by proper history taking, examination, and radiological study.

**Case Report:**

We reported the case of a seven-year-old female presenting with right-sided swelling in the neck aggravating in size while straining or coughing. Regular follow-up was advised. Swelling regression was observed after one year of follow-up without any surgical treatment.

**Conclusion:**

This is a self-limiting condition and usually the treatment is not warranted. Regular follow-up is advised for the patient.

## Introduction

Jugular vein ectasia means the dilatation of the jugular veins. It is commonly observed in children or young adults (1). Internal jugular vein (IJV) ectasia commonly presents as compressible swelling in the neck, soft in consistency, which typically aggravates in size with maneuvers that increase intrathoracic pressure, such as straining, Valsalva maneuver, sneezing or coughing and is usually unilateral. 

Differential diagnosis includes laryngocele, cystic hygroma, branchial cyst, and superior mediastinal mass (2). These conditions are usually observed in paediatric age group that result in a challenging diagnosis. Moreover, the incidence of this condition is rare and around 100 cases of phlebectasia have been reported in the English literature up to now in different neck veins, among which internal jugular is the most common to be affected followed by external jugular and anterior jugular veins(1,3). 

However, there is limited data mentioned in the literature regarding the regression of size without any intervention. Radiological studies are necessary to confirm the diagnosis and for records so that serial monitoring can be performed and documented. Chest X-ray can eliminate the diagnosis of laryngocele or mediastinal tumor as there will be the absence of air or wide mediastinum in the films, respectively. Diagnosis is confirmed by ultrasound combined with Doppler flow imaging or spiral computed tomography (CT) scan with contrast. Colour Doppler as a cheap technique is the diagnostic modality of choice, especially in developing countries. The treatment protocol is conservative in the absence of any complications or deformities (4).Surgical treatment is usually required for complications or for cosmetic purposes. Spontaneous rupture has not been reported up to now. 

## Case Report

**Fig 1:**
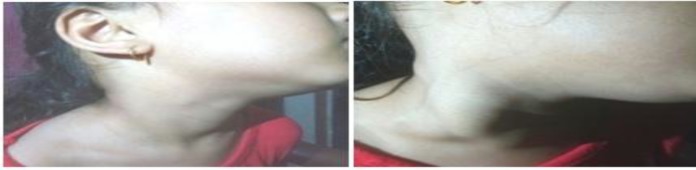
**A- **showing Photograph at rest **B-** showing Right sided neck mass appearing on valsalva

A seven-year-old girl presented in our OPD with right-sided neck swelling for four years, which increased while coughing or straining (Fig.1a,b).

It was insidious in the onset and progressively increasing. There was no significant medical history, no history of trauma, pain, fever, cough, weight loss, difficulty in swallowing, breathing or any other site swelling. In examination, there was a nonpulsatile, nontender, nontransilluminant, nonpulsatile, soft, and cystic swelling that increased in size by coughing. Moreover, other intrathoracic pressure raising manoeuvres was situated anteromedially to the right sternocleidomastoid muscle with the size of 4×3 cm. Nasal endoscopy and Fiber-optic laryngoscopic examination of the child was normal.

**Fig 2:**
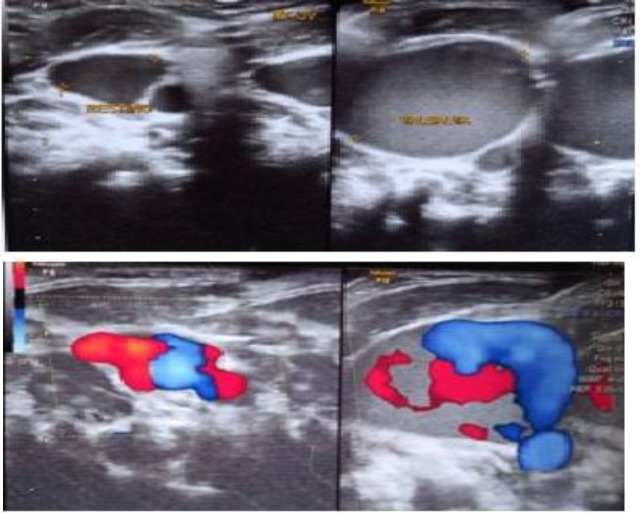
**A-** showing pre valsalva and post valsalva diameter of Right IJV on Ultrasound Neck.**B-** showing pre valsalva and post valsalva diameter of Right IJV on Color Doppler

Chest X-ray was grossly normal. Colour Doppler ultrasound was performed and finally internal jugular phlebectasia was diagnosed. On ultrasound, preValsalva diameter of IJV was 8.4 mm, while postValsalva diameter was 24 mm (Fig.2 a,b). 

Parents were counseled and reassured and no surgical treatment was offered as the patient was asymptomatic. 

**Fig 3:**
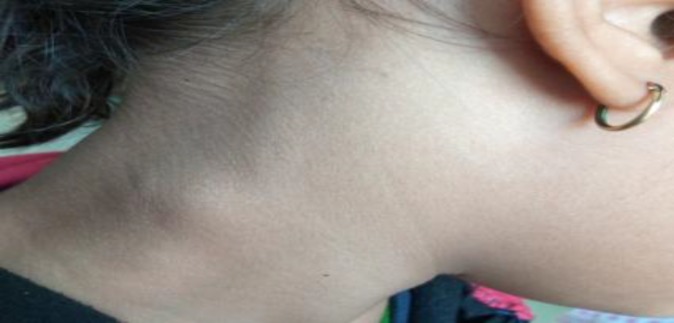
showing post valsalva swelling appearing on right side of neck after one year of follow up

**Fig 4:**
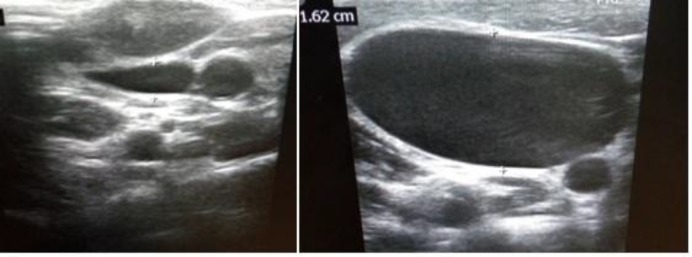
**A-** showing pre valsalva diameter of Right IJV on Ultrasound Neck. **B-** showing post valsalva diameter of Right IJV on Ultrasound Neck

The regular follow-ups were also suggested to parents with the advice of review at the earliest time if there was any change in the size of swelling and development of any symptoms. After one year of follow-up, swelling was observed to be decreased (Fig.3) and a repeated ultrasound was advised, which showed preValsalva diameter of IJV to be 6mm, as well as postValsalva diameter to be 16 mm, and confirmed decrease in the size of IJV (Fig.4a ,b). The parents were advised to visit ENT outdoor six monthly or any notice of increase in size. 

## Discussion

Phlebectasia is a condition that means the dilatation of vein without tortuosity. When there is dilatation with tortuosity, it is referred to as “varix”. Phlebectasia of the jugular veins presents as a soft cystic swelling in the neck during straining. For the first time, internal jugular phlebectasia was described by Zukschwerdt (5). The IJVs followed by external jugular veins are the most commonly affected veins. Our patient presented with right-sided swelling, which was supported by a hypothesis proposed by La Monte et al. (4)

Diagnosis is usually made using colour Doppler ultrasound as it confirms vascular flow (6). Contrast enhanced CT scans and venography are other diagnostic options. Typical clinical presentation was a child with soft, round or fusiform neck swelling located at the lower third of anterior border of the sternocleidomastoid muscle in the neck that increased in size with straining, coughing, bending, sneezing, Valsalva maneuver, or after exertion. The chief complaint was cosmetic. 

Color Doppler ultrasound is the investigation modality of choice as it is cost-effective. It can be easily and effectively used for monitoring the swelling so that the size can be documented. Therefore, ultrasound is the best screening method. However, contrast-enhanced computed tomography gives the exact extent of the venous abnormality. It should be differentiated from laryngocele, superior mediastinal cysts or other nonpulsatile masses of the neck (2).

Complications, such as thrombosis (7) and Horner’s syndrome (2), are reported; however, the incidence is very rare. Rupture has not been reported in the literature up to now. Our case had no such complications. In the absence of complications, the patient can be put under observation (8). We would like to highlight the role and importance of surveillance in such cases of phlebectasia of the neck veins that rarely causes any significant problems and can regress on its own. Furthermore, the surgery similar to this case should not be the prime target of the clinician. 

Surgery in such benign cases is usually reserved for complications. Surgical option includes the resection of dilated portion of vein or sheathing of the affected segment in a polytetrafluoroethylene tube graft (9). Jugular vein ligation is a too radical procedure for such a benign condition and definitely cannot be applied in cases with bilateral affliction (10). 

## Conclusion 

The IJV ectasia is a rare entity which presents in pediatric patients usually with the neck mass aggravating in size with maneuvers, such as coughing, straining or Valsalva. Imaging is essential for the diagnosis. Surveillance is the mainstay in most of the cases. It can regress spontaneously so it should be waited and watched. Surgery is suggested in the presence of complications or achieving cosmesis.
